# One-Pot Synthesis of Colloidal Hybrid Au (Ag)/ZnO Nanostructures with the Participation of Maleic Acid Copolymers

**DOI:** 10.3390/polym15071670

**Published:** 2023-03-27

**Authors:** Nadezhda A. Samoilova, Maria A. Krayukhina, Alexander A. Korlyukov, Zinaida S. Klemenkova, Alexander V. Naumkin, Yaroslav O. Mezhuev

**Affiliations:** 1 A. N. Nesmeyanov Institute of Organoelement Compounds, Russian Academy of Sciences, 28 Vavilova St., 119991 Moscow, Russia; 2 Department of Biomaterials, Mendeleev University of Chemical Technology of Russia, 9 Miusskaya Square, 125047 Moscow, Russia

**Keywords:** maleic acid copolymers, Au/ZnO and Ag/ZnO nanoparticles, one-pot synthesis

## Abstract

One-pot synthesis of colloidal Au/ZnO and Ag/ZnO nanohybrid structures was carried out. The copolymers of maleic acid—poly(N-vinyl-2-pyrrolidone-*alt*-maleic acid), poly(ethylene-*alt*-maleic acid), or poly(styrene-*alt*-maleic acid) were used as templates for the sorption of cations of metals-precursors and stabilization of the resulting nanoheterostructures. Simultaneous production of two types of nanoparticles has been implemented under mild conditions in an aqueous alkaline medium and without additional reagents. Equimolar ratios of the metal cations and appropriate load on all copolymers were used: molar ratio of maleic acid monomeric units of copolymer/gold (silver)cations/zinc cations was 1/0.15/0.23 (1/0.3/0.15). The process of obtaining the heterostructures was studied using UV-Vis spectroscopy. The kinetics of the formation of heterostructures was influenced by the nature of the maleic acid copolymer and noble metal cations used. A high reaction rate was observed in the case of using zinc and gold cations-precursors and a copolymer of maleic acid with N-vinylpyrrolidone as a stabilizer of nanoparticles. The structure of the synthesized polymer-stabilized heterostructures was studied using instrumental methods of analysis—XPS, FTIR, PXRD, and TEM. Under the conditions used, stable colloidal solutions of heterodimers were obtained, and such structure can be converted to a solid state and back without loss of properties.

## 1. Introduction

Lately, a focus has been on the conjugation of two or more nanomaterials to achieve increased multifunctionality as well as creating opportunities for the next generation materials (nanohybrids) with enhanced performance [1,2]. Among a variety of hybrid heterostructured materials, polyelement nanoparticles and metal-metal oxide heterostructures are widely studied and applied materials [2,3,4]. Great interest is caused by composites based on zinc oxide and metals—silver or gold. Such hybrid nanostructures have received great research attention because they combine the unique physical and chemical properties of the ingredients that make up the composite, owing to which a wide variety of areas of their use are possible. Zinc oxide nanoparticles are characterized by non-toxicity, biosafety, excellent biological compatibility, high electron transfer rate, good analytical characteristics, and low cost. Zinc oxide is a kind of wide gap semiconductor (3.37 eV) [5,6]. The inclusion of silver or gold nanoparticles in the heterostructures introduces additional properties such as high catalytic, optical sensitivity, and bactericidal activity. These nanoparticles possess universal biocompatibility, low toxicity, and high chemical stability [7]. So, the overlap of the spectral range of the incident photon with absorbance wavelength of the semiconductor (ZnO) and the surface plasmon bands of the plasmonic metal (Ag, Au) provides a useful tool to predict the enhancement in optical and electrical properties of hybrid semiconductor-noble metal nanostructures [8,9,10]. Many publications are devoted to the use of Au (Ag)/ZnO hybrid structures as photocatalysts [11,12,13,14,15]. This enhanced photoactivity of Au/ZnO composite materials was resultant from formation of stable and effective Schottky contact between metal and ZnO surfaces [16]. The photochemical activity of such heterostructures was used also to synthesize a number of organic compounds [17,18]. In addition, zinc oxide/gold (silver) heterostructures with plasmonic-enhanced photoelectrochemical activity also were used for photoelectrochemical hydrogen generation [19,20]. On the basis of heterodimeric composites, sensor systems were created [21,22,23,24,25,26]. Moreover, synthesis of ZnO nanostructures and their functionalization by Au nanoparticles for improved photocatalytic and high performance Li-ion battery anodes were carried out [27]. A large number of studies are devoted to the use of heterostructures mainly contained silver particles in biomedicine. Enhanced wound healing activity of Ag/ZnO composites was detected [28]. Virus-like mesopore silica-zinc oxide/Ag nanoparticles collected on NIR laser irradiation with quercetin were used to improve the elimination the mutated COVID-19 virus [29]. It is shown that Ag/ZnO complexes and various composites based on them have antibacterial activity [30,31,32], including activity against antibiotic-resistant pathogens [33,34]. The methods of noble metal—zinc oxide heterostructure production include electrochemical deposition, sputtering, chemical reduction, sol–gel method, template method, hydrothermal/solvothermal method, and so on [35,36,37,38,39]. Metal—zinc oxide heterostructures are mostly obtained by hydrolysis and condensation of intermediates. Most often, the hydrothermal/solvothermal method includes the use of reducing agents, different ligands, or surfactants. Obtaining such products is usually a two-step process, requiring high temperatures and autoclaving at the stage of formation and inclusion of zinc oxide. In this case, as a rule, conglomerates of nanoparticles are formed [17,19,28,33,40,41]. It seems attractive from the point of view of the simplicity of obtaining and further use of the one-pot method for the synthesis of Ag (Au)/ZnO heterodimers. Very few reports, in fact, are available regarding the single step synthesis. One-pot synthesis of the solid composite Ag(Au)/ZnO was carried out at high temperature in the presence of hydrazine hydrate [34], hexamethylenetramine [42], oleylamine [12,43]. High temperature one-pot non-aqueous solvothermal synthesis of Au/ZnO hybrids was reported by Zhang [36]. Chemical vapor deposition of modified Ag/ZnO composite was prepared by hydrothermal method, and then annealing was carried out at 600 °C [23]. Ag/ZnO composites were synthesized also using the “one-pot” solid-state pyrolysis method by co-heat-treatment of mixed Ag and Zn precursors at high temperature [44].

So, most of the processes of Ag(Au)/ZnO composites preparations including one-pot methods use organic media, high temperature and high pressure, long synthesis time and additional reaction agents. Most of these techniques do not lead to the production of water-dispersed colloids because of the spontaneous aggregation of clusters. The mentioned facts limit the use of such composites in various applications. To meet industrial, biomedical and another needs, a simple, inexpensive, and fast method of synthesis of Ag(Au)/ZnO colloidal nanocomposites is still required. The aim of this work is to develop a new very simple approach to one-pot synthesis of colloidal Ag(Au)/ZnO hybrid nanostructures, in the absence of additionally introduced reagents; in our case, both noble metal and zinc oxide nanoparticles are formed in the presence of a polymer matrix in an aqueous medium.

## 2. Materials and Methods

### 2.1. Materials

Poly(ethylene-*alt*-maleic anhydride) with an average molecular weight M = 25,000 and poly(styrene-*alt*-maleic anhydride) M = 50,000 were purchased from Monsanto (Saint Louis, MO, USA) and Sterlitamak chemical plant (Sterlitamak, Russia), respectively. Poly(N-vinyl-pyrrolidone-*alt*-maleic anhydride) M = 40,000 was prepared following a procedure described previously [45]. The copolymers before use were hydrolyzed to the corresponding copolymers of maleic acid (EM, SM, and VM) by dissolving in deionized water followed by lyophilization. The reagents NaBH_4_, HAuCl_4_·3H_2_O, and ZnSO_4_·7H_2_O (Sigma Aldrich, Munich, Germany) were used. AgNO_3_, NaOH (all of the analytical grade, Reahim, Moscow, Russia), were used without purification. VM stabilized nanosilver (VM/Ag^0^) and VM stabilized nanogold (VM/Au^0^) were prepared according to our approach [46].

### 2.2. Instrumentation

The pH values were determined using Fisher Scientific 300 403.1 pH-meter (Waltham, MA, USA). The UV-visible absorption spectra were obtained on UVIKON-922 spectrophotometer (Germany). A 0.2 cm cuvette was used for spectrophotometric measurements; registration was carried out without dilution of the reaction solution. Transmission electron microscopy (TEM) micrographs were obtained on a LEO 912 AB microscope (Omega, Karl Zeiss; Jena, Germany) operated at an accelerating voltage of 100 kV. For TEM observations, a drop of colloid solution was placed onto Formvar-coated copper grid, and then evaporated. FTIR spectra (KBr) were recorded on Fourier-spectrometer Magna IR-720 (Nicolet, Parsons W118, USA). The silver and gold content was determined by non-destructive X-ray fluorescence analysis (VRA-30 X-ray fluorescence spectrometer (Karl Zeiss, Jena, Germany). Powder XRD phase studies of samples were carried out with a D8 Advance (Bruker AXS, San Jose, CA, USA) diffractometer in the Bragg-Brentano focusing geometry using CuKα radiation, angular step was 0.02o, and the scan rate was 0.5 deg·min^−1^. The samples were placed on flat holders. Patterns were processed with DIFFRACplus EVA (Bruker AXS GmbH DIFFRAC.EVA, Bruker AXS GmbH, Karlsruhe, Germany, 2011). Bruker AXS software (Coelho, A. TOPAS 5.0, Bruker AXS GmbH, Karlsruhe, Germany, 2012) has Search/Match procedure implemented. The composition of samples was calculated with DIFFRAC TOPAS software (Coelho, A. TOPAS 5.0, Bruker AXS GmbH, Karlsruhe, Germany, 2012). Bruker AXS Rietveld refinement of collected X-ray data. The values of mean crystalline size were evaluated using Lvol-IB formalism [47]. X-ray photoelectron spectra were acquired with an Axis Ultra DLD (Kratos, Manchester, UK) spectrometer using monochromatized Al Kα (1486.6 eV) radiation at an operating power of 150 W of the X-ray tube. Survey and high-resolution spectra of appropriate core levels were recorded at pass energies of 160 and 40 eV and with step sizes of 1 and 0.1 eV, respectively. Sample area of 300 μm × 700 μm contributed to the spectra. The samples were mounted on a sample holder with a two-sided adhesive tape, and the spectra were collected at room temperature. The base pressure in the analytical UHV chamber of the spectrometer during measurements did not exceed 10−8 Torr. The energy scale of the spectrometer was calibrated to provide the following values for reference samples (i.e., metal surfaces freshly cleaned by ion bombardment): Au 4f_7/2_–83.96 eV, Cu 2p_3/2_–932.62 eV, Ag 3d_5/2_–368.21 eV. The electrostatic charging effects were compensated by using an electron neutralizer. The surface charge was taken into account according to the C–C/C–H state identified in the C 1s spectrum, to which a binding energy of 285.0 eV was assigned. After charge referencing, a Shirley-type background with inelastic losses was subtracted from the high-resolution spectra. The surface chemical composition was calculated using atomic sensitivity factors included in the software of the spectrometer corrected for the transfer function of the instrument.

### 2.3. Methods

#### Synthesis of ZnO/Au and ZnO/Ag Heterodimers

Synthesis of VM/Au/ZnO. Initially, a fixed quantity of solutions in deionized water: VM (20 mL, 0.007M, pH 8), ZnSO_4_·7H_2_O (0.322 mL, 0.1 M), HAuCl_4_·3H_2_O (2.1 mL, 0.01 M), and NaOH (0.4 mL, 1 M) were placed in test tubes with a screw caps tightly closed glass bottle. pH of obtained solution was 12. After shaking, the reaction mixture was placed in a bath with boiling water and kept for 3 h. The reaction solution was then stored at room temperature. The dried sample was obtained after dialysis (or ultrafiltration—YM5 membrane, “DIAFLO”, AMICON CORPORATION) of the reaction mixture with subsequent lyophilic drying (−55 °C, 0.05 mbar).

EM/Au/ZnO and SM/Au/ZnO samples were prepared under similar reagents concentrations, similar reaction conditions, and at the same molar ratio of monomeric units of maleic acid residues of copolymer/gold cations/zinc cations 1/0.15/0.23. The reaction time for these samples was 4 h.

Synthesis of VM/Ag/ZnO. The solutions in deionized water: VM (25 mL, 0.007 M, pH 8), ZnSO_4_·7H_2_O (0.262 mL, 0.1 M), AgNO_3_ (0.52 mL, 0.1 M), and NaOH (0.325 mL, 1 M) were placed in test tubes with screw caps. pH of obtained solution was 12. After shaking, the reaction mixture was placed in a bath with boiling water and kept for 6 h.

EM/Ag/ZnO and SM/Ag/ZnO samples were obtained under similar reagents concentrations, similar reaction conditions, and at the same molar ratio of monomeric units of maleic acid residues of copolymer/silver cations/zinc cations 1/0.3/0.15. The reaction time for these samples was 7 h.

## 3. Results and Discussion

To obtain stable colloidal nanostructured metal/metal oxide samples, the choice of the initial polymer matrix is important. The polymer matrix should serve as a suitable nanoparticles (NPs) coating, preventing their aggregation by reducing their surface energy. The stabilizing effect of macromolecules depends on the polymer structure, which makes it possible to carry out steric and (or) Coulomb stabilization of nanoparticles. It is also desirable to have functional groups in macromolecules for interaction with precursor-metal cations by forming a complex or an ion pair. The copolymers of maleic acid namely, poly (N-vinyl-2-pyrrolidone-*alt*-maleic acid) (VM), poly(ethylene-*alt*-maleic acid) (EM), or poly(styrene-*alt*-maleic acid) (SM) were used as templates for the sorption of cations of metals and stabilization of nanoheterostructures. These copolymers have a number of advantages: in addition to commercial availability or simple synthesis by radical copolymerization (in the form of copolymers of maleic anhydride), they have regular structure of macromolecular chains. The copolymers are amphiphilic and water-soluble. The process of forming colloidal heterostructures containing zinc oxide and gold (silver) nanoparticles stabilized by maleic acid copolymers can be divided into two successive stages: (1) formation of salts (complexes) of zinc and silver (gold) cations with copolymers of maleic acid; (2) hydrothermal matrix-dependent transformation of metal cations into zinc oxide nanoparticles and silver (gold) nanoparticles and their stabilization. In obtaining the target colloidal structures, the nature of the polymer played a key role at all stages of this process.

At the first stage, dicarboxylic acid residues having two closely spaced donor carboxyl groups formed an ionic or coordination-ionic bond with metal cations with valence or coordination number ≥ 1. At this stage, an alkaline medium (pH 8) was used in which polyacids are less associated and have a more expanded conformation of macromolecular chains, which should contribute to a statistically uniform distribution of metal cations in the polymer matrix [48]. At elevated pH, sensitivity to divalent cations, including zinc, attributed to the stabilization of the deprotonated maleic acid functionality [49,50,51]. It was shown that SM with different amounts of ZnCl_2_ resulted in SM-Zn ionomers formation with different degrees of chelation, and at the same time a rigid “pseudo-ring” structure is obtained [52]. We have previously shown [48,53,54] that one structural unit of the maleic acid residue of copolymers binds silver ion with the formation of a silver/dicarbonic acid complex of two coordination type; in this case, the process of binding silver cations in the alkaline environment occurs statistically (not cooperatively). So, Ag^+^ ion is coordinated with both carboxyl groups of the monomeric unit of maleic acid residues by a coordination-ionic bond in accordance with the coordination number of silver equal to 2 [48,53,54]. The binding of gold cations by dicarboxylic acid copolymers was studied by dialysis method [55]. The degree of binding of gold (Au^3+^) cations to the copolymers used was determined. At pH 10, the copolymers sorbed EM—36, SM—39, VM—46% (mol) of the metal cation. In our work, the load metal cations on copolymers did not exceed 30%. The presence of polar residues of N-vinylpyrrolidone in the structure of the VM copolymer can affect the binding of metal cations. It is known that polyvinylpyrrolidone (PVP) can coordinate metal cations due to the high affinity of pyrrolidone residues [56,57,58]. Nevertheless, it was shown that the interaction (coordination) of PVP with main metal cations is weak [59,60]. The degree of binding of PVP gold cations at pH 10 determined by our approach [55] did not exceed 13%. In this paper, we used equimolar ratios of zinc and noble metal cations. The load of cations on one maleic acid residues of the copolymers was Zn^2+^/Ag^+^ = 0.15/0.3 (mol/mol) for preparation of composites ZnO/Ag^0^ and Zn^2+^/Au^3+^ = 0.23/0.15 for ZnO/Au^0^. Heavy loads of metal cations on copolymers led to obtain less stable colloidal systems. In an aqueous alkaline medium, the hydrolysis of zinc, silver or gold salts occurred with the formation of corresponding metal cation hydroxides, which, in turn, can form chelate complexes with dicarboxylic acid residues and polar copolymer groups. The stage of transformation of metal cations into zinc oxide nanoparticles and gold (silver) nanoparticles took place in an alkaline medium and using a boiling water bath for reaction vessel. It is known that gold and silver cations are strong oxidizers (electrode potentials in the conditions of nanoparticle synthesis (T = 373 K, pH = 12) are calculated in Supplementary Materials (Calculated data) and make up for Au(OH)_3_, H^+^/Au and Ag_2_O, H^+^/Ag +0.56 V and +0.285 V, respectively). With a value of −237 kJ mol^−1^ for the Gibbs free energy of water, the standard reduction potential for the reaction 2H_2_O = O_2_ + 4H^+^ + 4e is 1.23 V. Under the conditions of nanoparticle synthesis, the electrode potential of the last half-reaction is significantly less than under standard conditions and is +0.34 V. Because this value is lower than that of 1.50 V for the Au^3+^—Au^0^ reaction, Au^3+^ is unstable in water, and will undergo spontaneous reduction. In an alkaline medium, the gold salt is hydrolyzed to the corresponding hydroxide, and then the target product appears: Au(OH)_3_ + 3H^+^ + 3e = Au^0^ + 3H_2_O [61]. Similarly, in an alkaline medium, Ag^+^ passes into silver hydroxide and then silver (I) oxide. However, for the reduction of silver (I) oxide (T = 373 K, pH = 12), water is ineffective, since the electrode half-reaction potential of Ag_2_O, H^+^/Ag^0^ is +0.285 V. At the same time, Ag_2_O has a small absolute value of the standard Gibbs energy of formation −11.3 kJ/mol. As shown in Supplementary Materials (Calculated data), taking into account the influence of dispersion leads to a positive Gibbs energy of the formation of silver oxide (I) nanoparticles with a diameter of the order of several nanometers. Thus, we can expect spontaneous decomposition of silver oxide in the composition of nanoparticles by the reaction Ag_2_O = 2Ag^0^ + 0.5O_2_. At the end of the reaction, the acidification of the reaction medium was observed—from pH 12 to 9–10. The nature of the zinc bond in the compounds also changes from ionic in the initial salt to covalent in the oxide during the reaction: ZnSO_4_ → Zn(OH)_2_ → ZnO. The chemical bond between zinc and oxygen in ZnO molecule is predominantly covalent but with a significant contribution from ionic bonding [62]. It was previously shown that under heating at 150 °C for 3 days of SM-Zn polychelate, a small amount of ZnO nanospheres was detected [52]. In our case this process, apparently, was stimulated in an alkaline environment and in the presence of gold (silver) cations.

**Figure 1:**
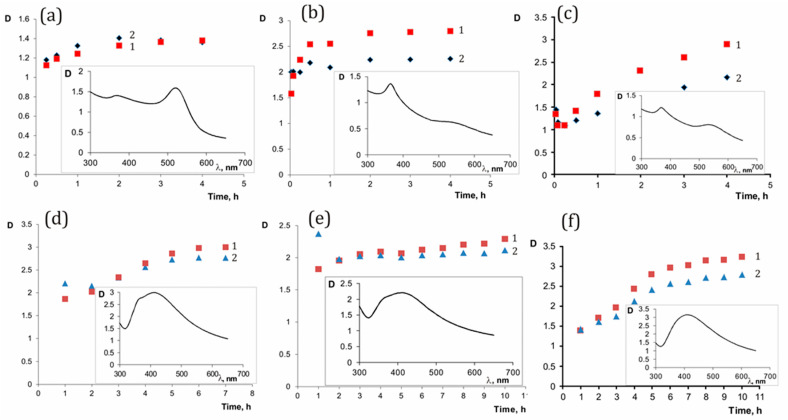
UV-Vis spectra and the kinetics of formation of heterodimers (95 °C, pH 12): VM/Au^0^/ZnO (**a**), EM/Au^0^/ZnO (**b**), SM/Au^0^/ZnO (**c**), VM/Ag^0^/ZnO (**d**), EM/Ag^0^/ZnO (**e**), and SM/Ag^0^/ZnO (**f**). Curves 1—the change in optical density at 406 or 520 nm for plasmon resonance nanoparticles Ag^0^ or Au^0^, respectively; curves 2—the change in optical density at 370 nm for zinc oxide during the reaction. Inset: the UV-Vis spectra of the final products of the reactions.

The reaction products formation in the system can be tracked by changes in the UV-Vis spectra due to specific absorption bands of reaction products [25,63,64]. Figure 1 shows the UV-Vis spectra illustrating the kinetics of the formation of ZnO and Au^0^ or Ag^0^ nanoparticles, which are components of heterodimers. 

The ZnO/Au^0^ and ZnO/Ag^0^ nanocomposites exhibit strong absorption in the visible region as shown in Figure 1. The absorption wavelength or broad band around 365–375 nm was the specific peak of ZnO, absorption peaks at 520 nm and band at 406 nm were attributed to the Au^0^ and Ag^0^ nanoparticles, respectively [64,65,66]. At the initial moment of the reaction, turbidity was observed in the system, apparently due to the formation of zinc hydroxide (zinc hydroxide polymer derivatives). Then, during the reaction, the system becomes more transparent and acquires a color characteristic of gold or silver nanoparticles caused by their surface plasmon resonance. At the same time, the absorption band characteristic of zinc oxide also increased, and changed almost synchronously with the change in the absorption band of the nanometal, except for the initial period of time (Figure 1a–f). The obtained dilute solutions of heterodimers are shown in Figure S1 (Supplementary Materials). In the presence of gold and zinc cations and a VM stabilizer copolymer under the selected conditions, the reaction was almost completed in 2 h, for SM and EM it took about 3–4 h (Figure 1a–c), while the characteristic absorption bands of the final products were finally formed and their intensity practically did not change. In the presence of gold and zinc cations and the copolymer-stabilizer VM under the selected conditions, the reaction was practically completed after 2 h, for SM and EM it took about 3–4 h (Figure 1a–c), while the characteristic absorption bands of the final products were finally formed and their intensity practically did not change. The test for the presence of residual noble metal ions at the end of the reaction was carried out for gold ions. For this purpose, we investigated the changes in the absorbance of silver nanoparticles during the galvanic oxidation of these nanoparticles with perhaps present gold cations from our samples [55,67]. In this case, the working solution was a copolymer of ethylene with maleic acid containing silver nanoparticles (EM/Ag^0^), obtained and characterized by our research group earlier [54]. The optical density of the initial nanosilver solutions changes slightly with the introduction of Au-containing heterodimers (Figure S2). So, quality test showed the negligible amount of gold cations in the system after the specified reaction time. For a heterodimer containing zinc oxide, a specific luminescence was recorded (Figure S3). In the case of using of the silver cations instead of gold cations in the reaction of heterostructure preparation, the trend persisted, but the process lasted longer. For the copolymer VM, the reaction was almost over after 6 h, for SM and EM—in about 7 h (Figure 1d–f). The high reaction rate of the formation of heterodimers when using gold cations is associated with their more pronounced oxidizing properties. The maleic acid copolymers used have a similar molecular weight; however, the reaction rates in the presence of copolymers of maleic acid with different comonomers differ. A noticeable increase in the rate of heterodimer formation in the presence of the VM copolymer is apparently due to the presence of pyrrolidone residues in its structure, which can affect the binding of cations and the process of their nucleation [59,60]. Under our reaction conditions of obtaining heterodimers the use of PVP instead of maleic acid copolymers resulted in a black precipitate after 15 min, although PVP is known to be a stabilizing agent and a weak reducing agent for Au^3+^ and Ag^+^ [68,69]. The control experiment with involving in reaction system cations of noble metal and zinc in the absence of a polymer matrix resulted in unstable systems with product precipitation.

**Figure 2:**
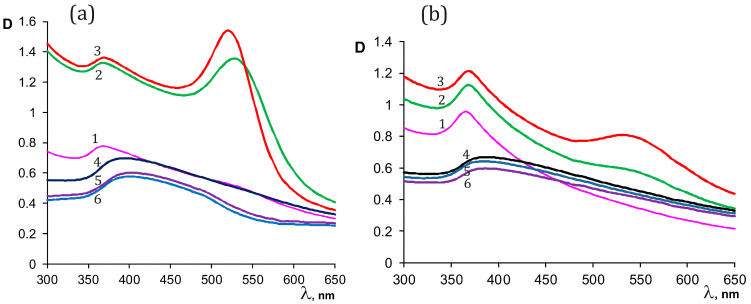
UV-Vis spectra of systems VM/Au^3+^/Zn^2+^ (**a**) and SM/Au^3+^/Zn^2+^ (**b**) under molar ratio of copolymer maleic acid residues/gold cations/zinc cations 1/0.15/0.23 (1–2 min, 2–1 h, 3–4 h) and VM/Zn^2+^ (**a**) and SM/Zn^2+^ (**b**) under molar ratio of copolymer maleic acid residues/zinc cations 1/0.23 (4–2 min, 5–1 h, 6–4 h); 95 °C, initial pH 12.

Figure 2 shows the evolution of the optical spectra in the preparation of the heterodimers VM/Au^3+^/Zn^2+^ (Figure 2a (1–3)) and SM/Au^3+^/Zn^2+^ (Figure 2b (1–3)), and the spectra of reaction products in the polymer-zinc cations systems in the absence of gold (Figure 2a,b, (4–6)). During the synthesis of heterodimers, an increase in optical density is observed in the regions of 520 and 370 nm, which are characteristic of gold and zinc oxide, respectively (Figure 2a,b, (1–3)).

Interestingly, under conditions similar to those used in the production of heterodimers (pH, temperature, copolymer-Zn cations ratio), but in the absence of gold cations, suspended particles were present in the system during the entire observation time. In this case nonspecific absorption was recorded, and the absorption was changing in time (Figure 2a,b (4–6)). A similar trend was observed in the case of the formation of heterodimeric structures VM/Ag^0^/ZnO, SM/Ag^0^/ZnO, EM/Au^0^/ZnO, EM/Ag^0^/ZnO, and copolymer/zinc oxide under the same conditions (Figure S4). Apparently, in the process of synthesis, macromolecules of copolymers acquire a conformation optimal for stabilizing the resulting nanoparticles. In the absence of forming silver (gold) nanoparticles, the conformation of macromolecules is not optimal for fixing zinc oxide nanoparticles. So, earlier [48] using the DLS method we showed that EM was characterized by an intermolecular association with a wide bimodal particle size distribution (R_h_ = 50–300 nm). For the corresponding nanosilver-containing complex, EM/Ag^0^ well-formed micelles, narrowly dispersed in size, were revealed (R_h_ = 105 nm). Silver (gold) nanoparticles come into contact with hydrophobic domains of the macromolecular chain, and polar zinc oxide particles tend to hydrophilic parts of macromolecules facing the external, aqueous medium. That is, under the conditions used, the copolymers, apparently, contribute to the synthesis of heterodimer nanocomposites due to conformational features of the structure of polymer–cation complexes at the initial stage, as well as steric and Coulomb stabilization of the resulting nanoproducts of the reaction due to the presence of contributing structural units and groups of amphiphilic copolymers.

**Figure 3:**
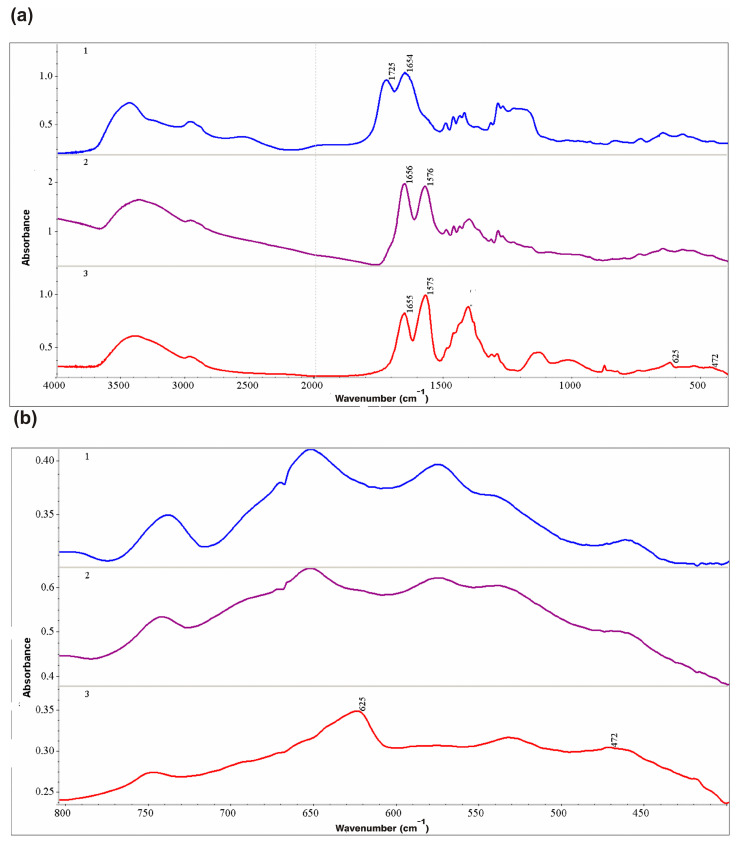
(**a**,**b**). FTIR spectra of VM (1), VM/Ag^0^ (2) and VM/Ag^0^/ZnO (3) (two wavelength ranges).

Figure 3a,b shows as an example the Fourier transform infrared (FTIR) spectra of the original VM copolymer, a copolymer containing nanosilver VM/Ag^0^ and VM/Ag^0^/ZnO heterostructure. In all spectra (Figure 3a), C=O stretching mode at 1654–1656cm^−1^ is consistent with a pyrrolidone copolymer ring [70,71].

A noticeable change in the spectrum of VM/Ag^0^ and VM/Ag^0^/ZnO, in contrast to that of VM, is associated with the position of the C=O bond of the carboxyl group. In the compound VM, C=O stretching vibration at 1725 cm^−1^ corresponds to non-ionized carboxyl groups of maleic acid residues. In VM/Ag^0^ and VM/Ag^0^/ZnO compounds, C=O stretching vibration at 1575–1576 cm^−1^ corresponds to the ionized form of carboxyl of the maleic acid residues of the copolymers since the copolymers are present in the form of a sodium salt [72]. For other polymers-stabilizers, this band is in the range 1560–1565 cm^−1^ (Figure S5a). Since the zinc oxide content in all the studied heterostructures is small (approximately 4–5%, weight), for greater clarity, the area of 400–800 cm^−1^ is presented in more detail in Figure 3b. The region 700–800 cm^−1^ shows the deformation non-planar oscillation of C-H bonds [72]. Occurrence of the at 472 cm^−1^ is characteristic of the formation of Zn-O bond (Figure 3b) [33,73]. Earlier for ZnO crystals, Zn-O stretching mode has also been observed in the range from 400 to 700 cm^−1^ [74,75,76]. Significant band at 625 cm^−1^ can be assigned to the Ag/ZnO nanocomposite formation (Figure 3b) [33,77].

**Figure 4:**
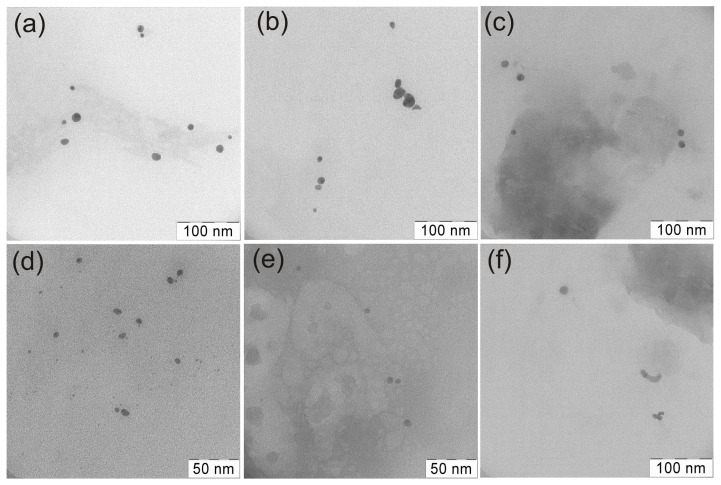
TEM micrographs of samples: VM/Au^0^/ZnO (**a**), EM/Au^0^/ZnO (**b**), SM/Au^0^/ZnO (**c**), VM/Ag^0^/ZnO (**d**), EM/Ag^0^/ZnO (**e**), SM/Ag^0^/ZnO (**f**).

Figure 4 depicts typical transmission electron microscopy (TEM) micrographs of the obtained composites nanoparticles.

Transmission electron microscope (TEM) investigation of colloidal solutions of synthesized polymer- stabilized Au/ZnO and Ag/ZnO showed relatively monodispersed nanoparticles. Similar crystalline nanostructures were found in all solutions of heterodimers prepared. Crystalline sizes for each phase are given also below (see XRD data). Some transparent and dark areas in the crystal particles were detected in the TEM image. Previously, using EDS analysis it was found that C, Zn, and O signals, but not Ag, were observed in more transparent particles, indicating that these particles are associated with ZnO. Peaks C, Zn, O, and Ag were detected at the interface between the dark and transparent regions [78]. Moreover, polar zinc oxide particles seem to gravitate toward polar fragments of chains of amphiphilic copolymers, and hydrophobic noble metal particles—toward their hydrophobic structural elements. Similar complexes (Au−PVP−ZnO) were demonstrated for polyvinylpyrrolidone [71]. Also earlier, the composite structure with ZnO surface modified with maleic acid, where the carbonyl groups of carboxylic acid were coordinated with the oxide metal [79].

**Figure 5:**
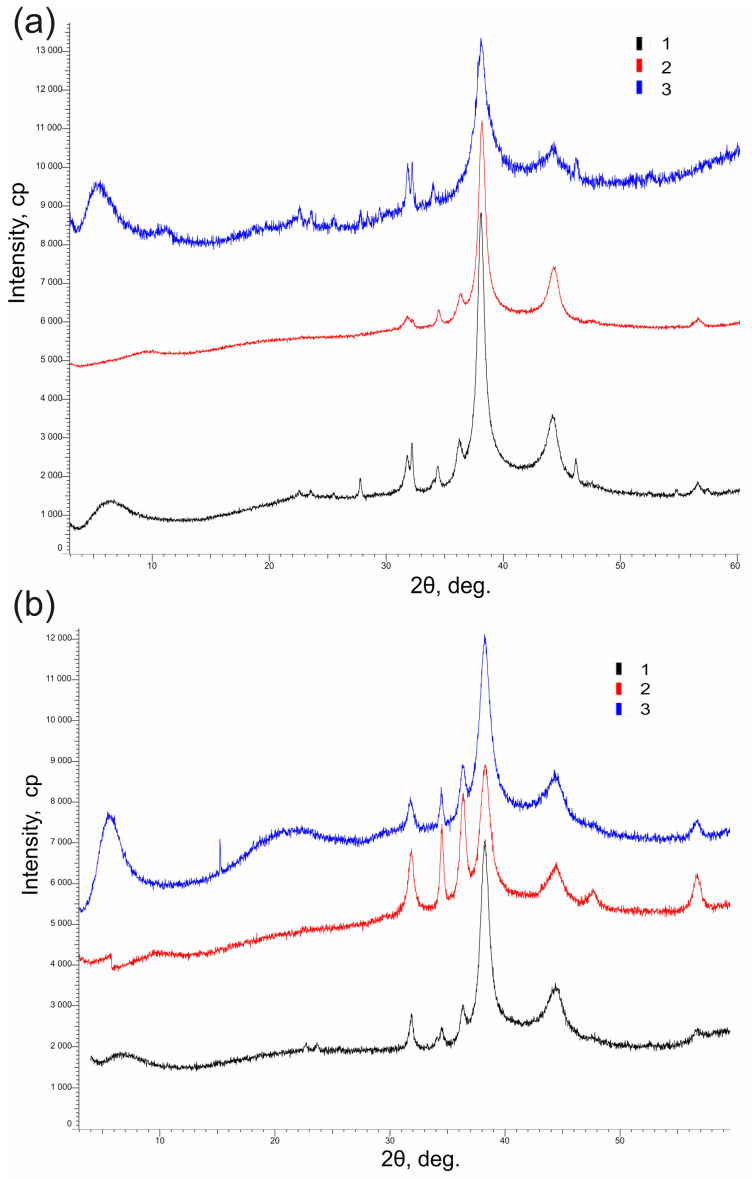
XRD patterns of heterodimers used: (**a**) VM/Ag^0^/ZnO (1), EM/Ag^0^/ZnO (2), SM/Ag^0^/ZnO (3); (**b**) VM/Au^0^/ZnO (1), EM/Au^0^/ZnO (2), SM/Au^0^ZnO (3).

The crystal structure of metal-containing constituents in the heterostructures studied by PXRD method (Table 1, Figure 5).

Figure 5 shows the XRD spectra of synthesized samples.

XRD studies of the analyzed samples have shown the presence of several different phases related to hexagonal phase of zincite (ZnO) and cubic phases of silver or gold as well. The peaks that correspond to Au phase are approximately: 38.2, 54.5, 64.7, and 78.7; Ag phase are approximately: 38.1, 44.2, 64.4, 78.3; ZnO (zincite phase) are approximately: 31.8, 34.4, 36.1, 47.5, 56.7, 63.0, 66.5, 68.0, 69.1, 72.6, 79.0°. The pattern of organic polymers is masked by the signal of inorganic and metal phases, and most likely it is merely scattering of amorphous phase. All phases found in samples can be described as nano-dimensional (Table 1). Indeed, the mean crystalline sizes of silver in corresponding samples was in the range of 6–9 nm, the size of the zincite crystals was 16–20 nm, and the content of nanosilver in the crystal phase was 79–92% and zinc oxide 8–20%. All gold-containing samples are characterized by almost the same size of gold nanoparticles varying in a narrow range 5–8 nm, the size ZnO crystals was 15–21 nm; preparations contained 62–78% nanometal and 22–38% zincite.

Comparison of XRD data with TEM and XPS (see below) results suggests the formation of polycrystalline heterostructures in the samples.

**Figure 6:**
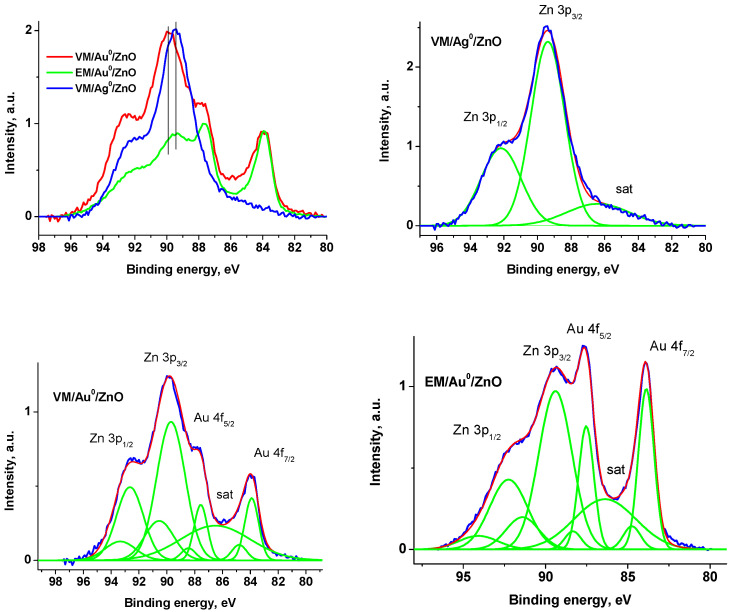
The Au 4f and Zn 3p high-resolution spectra of the VM/Au^0^/ZnO, EM/Au^0^/ZnO, and VM/Ag^0^/ZnO samples.

The chemical states of elements in the samples in the form of sodium salts were studied by XPS. The survey XPS spectra of the samples are displayed in Figures S6–S9. The high-resolution C 1s and N 1s spectra of the samples are shown in Figures S10 and S11, respectively. The quantification data derived from the survey and high-resolution spectra are presented in Table S1. As an example, a detailed analysis of the spectra of a number of heterodimers is given below. Figure 6 displays the Au 4f and Zn 3p spectra fitted with several Gaussian profiles. Table 2 shows their assignments and characteristics.

**Figure 7:**
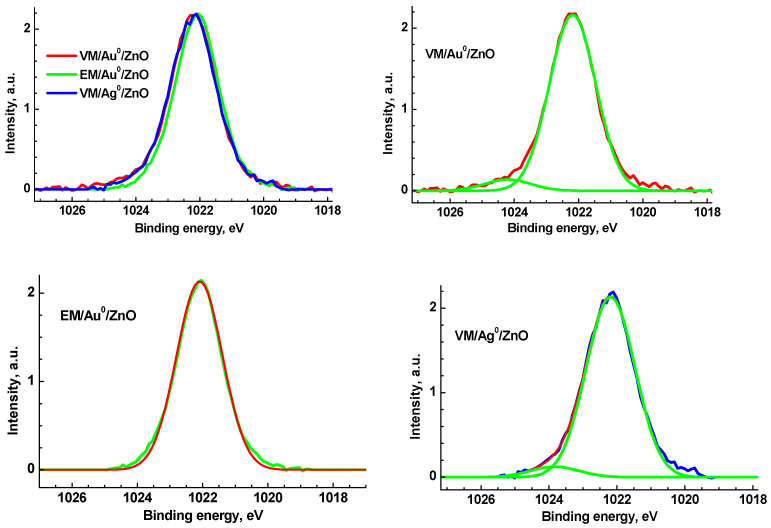
The Zn 2p_3/2_ high-resolution spectra of the VM/Au^0^/ZnO, EM/Au^0^/ZnO, and VM/Ag^0^/ZnO samples.

Figure 7 shows the Zn 2p_3/2_ spectra of the investigated samples.

The Zn 2p_3/2_ spectra of samples (Figure 7) are rather similar with a slight asymmetry in the high energy region and were fitted with two Gaussian peaks at 1022.2 and 1024.2 eV and 1022.2 and 1023.8 eV, respectively, with equal Gaussian widths of 1.44 eV. The spectrum of sample EM/Au^0^/ZnO sample is approximated with one Gaussian profile at 1022.1 eV and GW = 1.4 eV.

**Figure 8:**
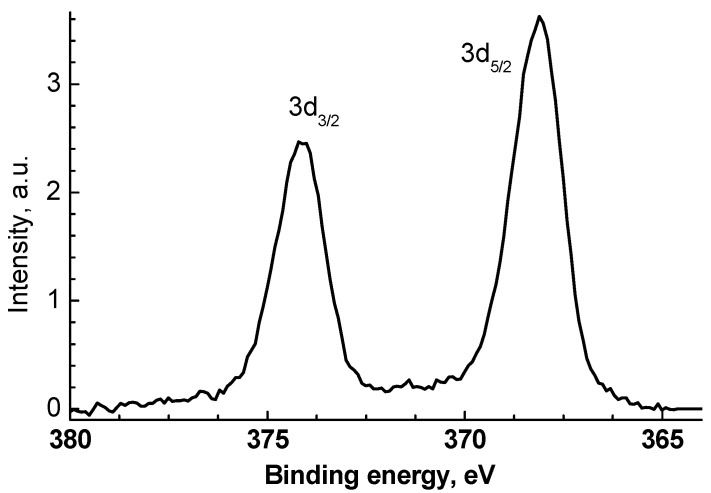
The Ag 3d high-resolution spectrum of the VM/Ag^0^/ZnO sample.

The Au 4f spectra were fitted with two spin-orbit doublets with 4f_7/2_-4f_5/2_ spin-orbit splitting of 3.65 eV and 3d_5/2_/3d_3/2_ branching ratio of 4/3. The Au 4f_7/2_ peaks located at ~83.9 and 84.1 eV are attributed to Au^0^ and Au^3+^ states that is in accordance with previous studies [80,81,82]. A small negative shift of 0.1 eV of the Au 4f_7/2_ peak compared to that of bulk Au (84.0 eV) may be ascribed to Au-Zn-O species, while a positive shift—to Au-O-Zn ones. The Zn 3p spectrum of VM/Ag^0^/ZnO sample was fitted with one spin-orbit doublet with 3p_3/2_-3p_1/2_ spin-orbit splitting of ~2.7 eV and 3p_3/2_/3p_1/2_ branching ratio of 2 and a peak at 86.4 eV which approximated to a low-energy tail of the spectrum and may be related to any specific loss. Such a tail was recorded in some other references as well [83,84,85]. The Zn 3p spectra of the VM/Au^0^/ZnO and EM/Au^0^/ZnO samples were fitted with two spin-orbit doublets with the same constraints. The fitting parameters of the Au 4f and Zn 3p spectra are presented in Table 2. The binding energies of Au 4f, Zn 2p_3/2_, and Zn 3p peaks of the AuZn-1 and AuZn-2 samples show some different AuZn species. The Ag 3d spectrum presented in Figure 8 is characterized with two peaks at 368.1 and 374.1 eV related to Ag 3d_5/2_ and Ag 3d_3/2_, which may be attributed to Ag^0^ state. As in case of Au 4f_7/2_, the slight negative shift by 0.2 eV relative to that of bulk sample (368.27 eV) [80] may be induced by Ag-Zn interaction. For Ag-doped ZnO [83], Ag 3d5/2 peak at 367.6 eV and Ag 3d_3/2_ peak at 373.6 eV were observed and assigned to Ag-O bonds. It should be noted that the Zn 2p_3/2_—Au 4f_7/2_ energy intervals for Ag-doped ZnO and VM/Ag^0^/ZnO are close, 654.1 and 654.2 eV, respectively. However, in the case of VM/Ag^0^/ZnO, there are signals after the Ag 3d_5/2_ and Ag 3d_3/2_ peaks, in which satellite peaks characterizing Ag^0^ can be inscribed, while they are not observed for Ag-doped ZnO.

Based on the data obtained, it can be assumed that the heterostructures obtained are core-shell structures, where metal nanoparticles can be located in the core, and zinc oxide is located in the outer layer [3].

## 4. Conclusions

We have demonstrated the one-pot hydrothermal synthesis of polymer-stabilized Au/ZnO and Ag/ZnO dimer nanostructures. The formation of stable colloids containing nanoforms of zinc oxide and gold (silver) was facilitated by the presence in the initial system simultaneously of two types of precursors—zinc cations and gold (silver) cations, as well as polymer matrices used. At the same time, the presence of a stabilizing polymer template is the determining factor in one-pot synthesis of colloidal polymer-stabilized Ag(Au)/ZnO hybrid nanostructures. The reaction rate of the formation of heterodimers depends on the nature of the stabilizing copolymer of maleic acid and noble metal cations introduced into the system. A high reaction rate was observed in the case of using the most hydrophilic copolymer of maleic acid with N-vinylpyrrolidone and gold cations, having a large electrode potential. The obtained heterodimer colloids are stable for at least six months, while the preparations can be dried for long-term storage with the possibility of subsequent dissolution and use. This cost-effective simple synthesis strategy can be useful as a platform to preparation of various metal—metal oxide nanostructured materials for in particular targeted biomedical applications for example, as contrast agents when introducing magnetite into their composition [4]. Bactericide-containing colloidal heterodimer nanomaterials may be use as antibacterial agents to address the ever-increasing problem of antibiotic-resistant bacteria [86]. At the same time, the presence of a stabilizing polymer in the obtained composites, which has easily modifiable carboxyl (anhydride) groups, makes it possible to introduce additional amine-containing antibiotic substances into the composition of the drug. The composites obtained can be converted into an insoluble form by crosslinking or by interpolyelectrolyte complexes formation [87], which will expand the range of their application.

## Figures and Tables

**Table 1:** The results of XRD studies of heterodimers.

Sample	Composition of Crystal Phase	Phase Amount, % (Deviation)	Crystalline Size for Each Phase, nm (Deviation)
VM/Ag/ZnO	Zincite, Silver	20.5 (6), 79.5 (6)	16.2 (8), 8.64 (8)
EM/Ag/ZnO	Zincite, Silver	19.2 (7), 80.8 (7)	12.9 (7), 7.76 (8)
SM/Ag/ZnO	Zincite, Silver	8.2 (10), 91.8 (10)	18 (4), 6.04 (10)
VM/Au/ZnO	Zincite, Gold	22.5 (17), 77.5 (17)	20.7 (16), 6.57 (6)
M/Au/ZnO	Zincite, Gold	37.6 (7), 62.4 (7)	17.9 (3), 7.91 (10)
SM/Au/ZnO	Zincite, Gold	35 (2), 65 (2)	15.0 (8), 5.05 (7)

**Table 2:** Characteristics of the Au 4f, Zn 2p, and Zn 3p photoelectron spectra: binding energies (Eb), Gaussian widths (W), and relative intensities (Irel) of photoelectron peaks belonging to different chemical groups in the C 1s and N 1s spectra.

Sample		Au 4f_7/2_	Au 4f_5/2_	Au 4f_7/2_	Au 4f_5/2_	Zn 2p_3/2_	Zn 2p_3/2_	Zn 2p_3/2_	Zn 3p_1/2_	Zn 3p_3/2_	Zn 3p_1/2_	sat	Zn 2p_3/2_ –Zn 3p_3/2_	Zn 2p_3/2_ –Au 4f _7/2_	Zn 3p_3/2_ –Au 4f _7/2_
VM/Au^0^/ZnO	E_b_, eV	83.9	87.6	84.8	88.5	1022.2	1024.2	89.7	92.7	90.6	93.4	86.5	932.5	938.2	5.8
	W, eV	1.0	0.96	1.05	1.03	1.44	1.44	2.05	1.94	2.1	2.2	5.0			
	I_rel_	0.46	0.33	0.12	0.09	0.94	0.06	0.52	0.26	0.15	0.08				
EM/Au^0^/ZnO	E_b_, eV	83.9	87.5	84.8	88.3	1022.1		89.4	92.3	91.4	94.1	86.4	932.7	938.3	5.5
	W, eV	0.95	1.0	1.05	1.0	1.41		2.03	2.3	2.01	2.39	3.75			
	I_rel_	0.49	0.37	0.08	0.06			0.55	0.28	0.11	0.06				
VM/Ag^0^/ZnO	E_b_, eV					1022.2	1023.8		89.4	92.2		86.4	932.8		
	W, eV					1.44	1.44		2.01	2.39		3.75			
	I_rel_					0.94	0.06		0.67	0.33					

## Data Availability

The data presented in this study are available on request from the corresponding author.

## Supplementary Materials

The following supporting information can be downloaded at: https://www.mdpi.com/article/10.3390/polym15071670/s1. Figure S1. The view of diluted solutions of samples. Figure S2. Test for the presence of gold cations in the products. Figure S3. Photoluminescence spectra of colloidal suspensions. Figure S4. UV-Vis spectra of reaction systems. Figure S5. FTIR spectra of samples. Figures S6–S9. The survey XPS spectra of heterodimers samples. Table S1. The quantification data derived from the survey and high-resolution spectra. Figure S10. The C 1s high-resolution spectra of the samples. Figure S11. The N 1s high-resolution. Figure S12. Calculated data.
