# Supplementary material for: One-Pot Synthesis of Colloidal Hybrid Au (Ag)/ZnO Nanostructures with the Participation of Maleic Acid Copolymers
Source: Polymers (Basel). 2023 Mar 27;15(7):1670. doi: 10.3390/polym15071670 (PMC10096674; doi:10.3390/polym15071670)
Supplement: Supplementary file 1 [file polymers-15-01670-s001.zip › polymers-2287599-supplementary.pdf]

Supplementary Materials

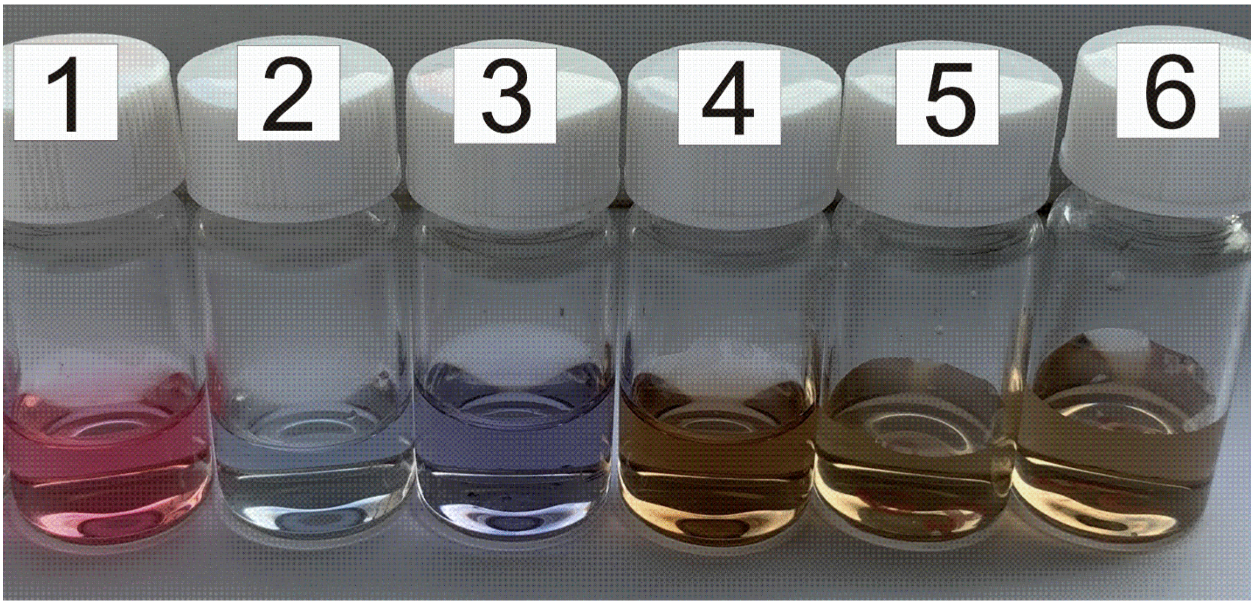

Figure S1. The view of diluted solutions of samples: 1-3 samples VM/Au^0/ZnO, SM/Au^0/ZnO and EM/Au^0/ZnO, respectively, at molar ratio 1/0.15/0.23; 4-6 samples VM/Ag^0/ZnO, SM/Ag^0/ZnO and EM/Ag^0/ZnO, respectively, at molar ratio 1/0.3/0.15

Test for the presence of gold cations in the products of the using the method of galvanic oxidation of silver nanoparticles [1] (Figure S2).

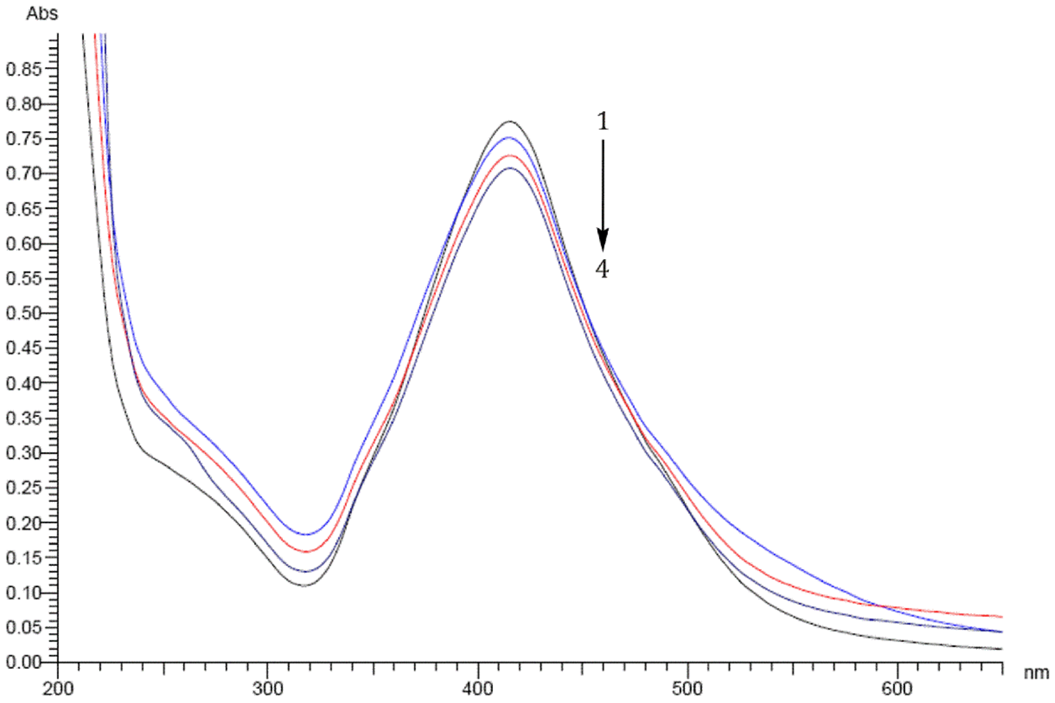

Figure S2. Test for the presence of gold cations in the products. 1 – control EM/Ag^0, 2 - 4 – EM/Ag^0 in the presence of VM/Au^0/ZnO, EM/Au^0/ZnO and SM/Au^0/ZnO, respectively. The deviation from the control is 3-9%.

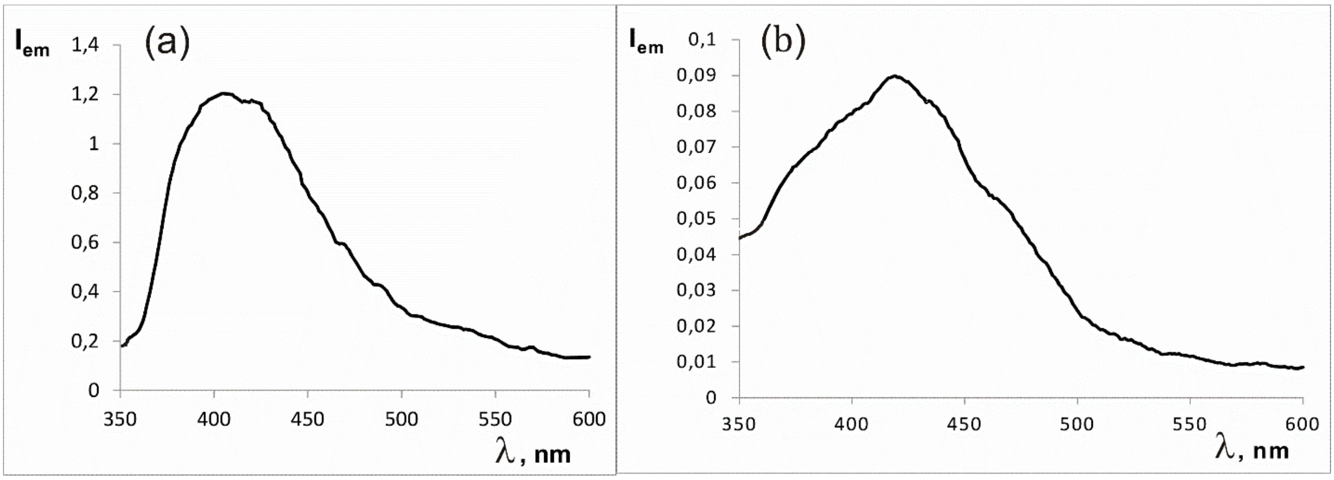

Figure S3. Photoluminescence spectra of colloidal suspensions ZnO (a) and VM/Au^0/ZnO (b) were produced through ablation in water (excitation by a 325 nm radiation [2].

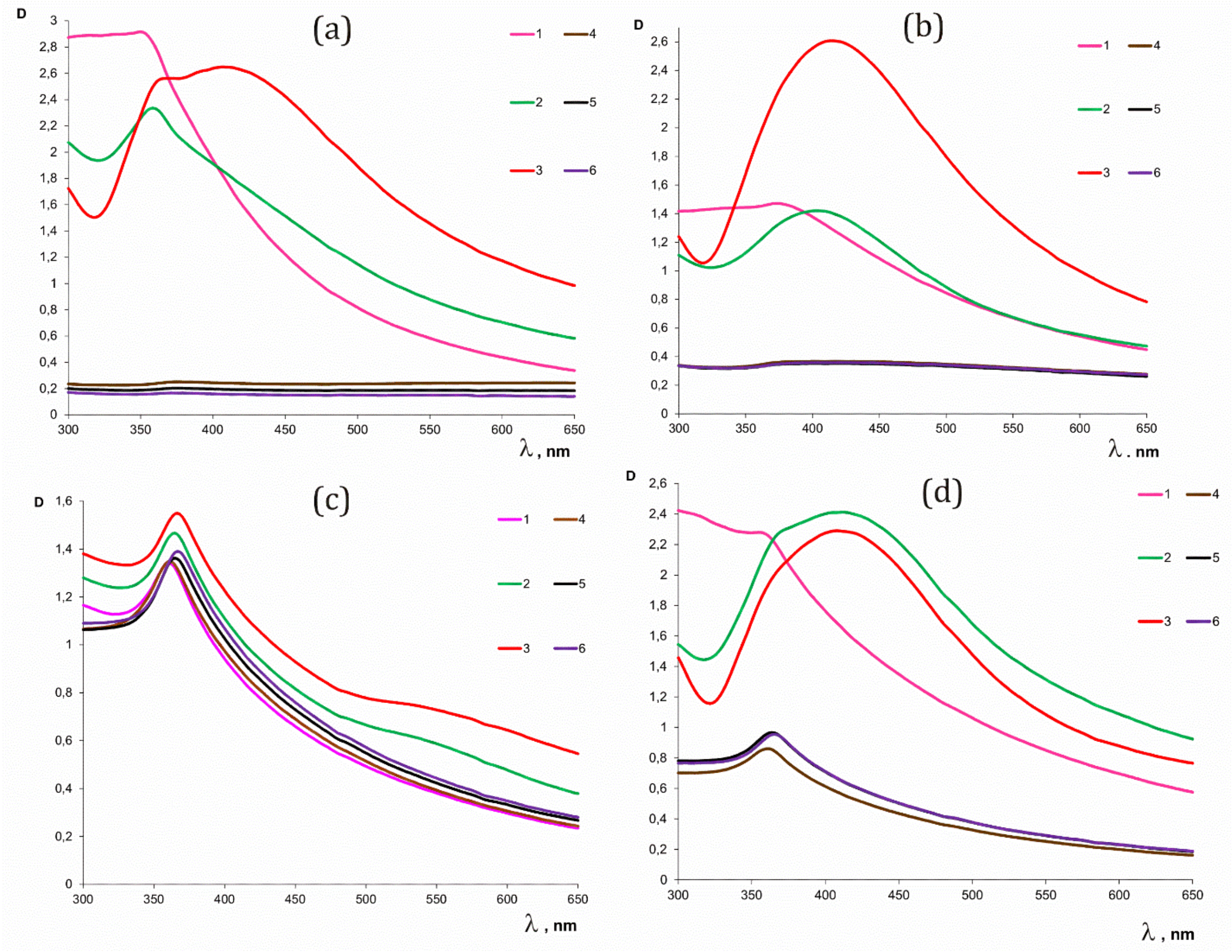

Figure S4. UV-Vis spectra of reaction systems: VM/Ag^+/Zn^{2+} (a), SM/Ag^+/Zn^{2+} (b) EM/Au^{3+}/Zn^{2+} (c) and EM/Ag^+/Zn^{2+} (d) under molar ratio of copolymer maleic acid residues/gold cations/zinc cations 1/0.15/ 0. 23, for silver cations molar ratio 1/0.3/0.15 (1 – 2 min, 2 – 1 h, 3 – 4 h) and VM/ Zn^{2+} (a), SM/ Zn^{2+} (b) and EM/ Zn^{2+} (d) under molar

ratio of copolymer maleic acid residues/zinc cations 0.3/0.15, for EM/ Zn^{2+} (c) ratio - 1/0.23 (4 – 2 min, 5 – 1 h, 6 – 4 h); 95°C, initial pH 12.

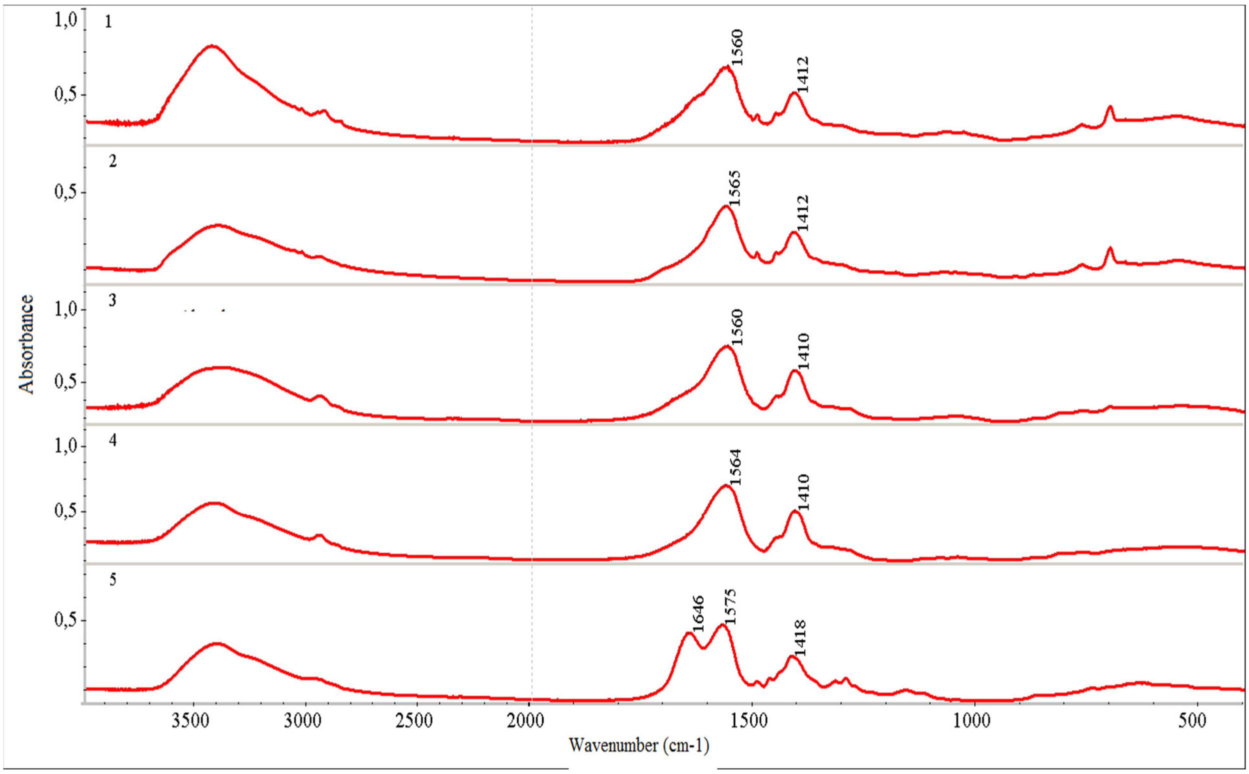

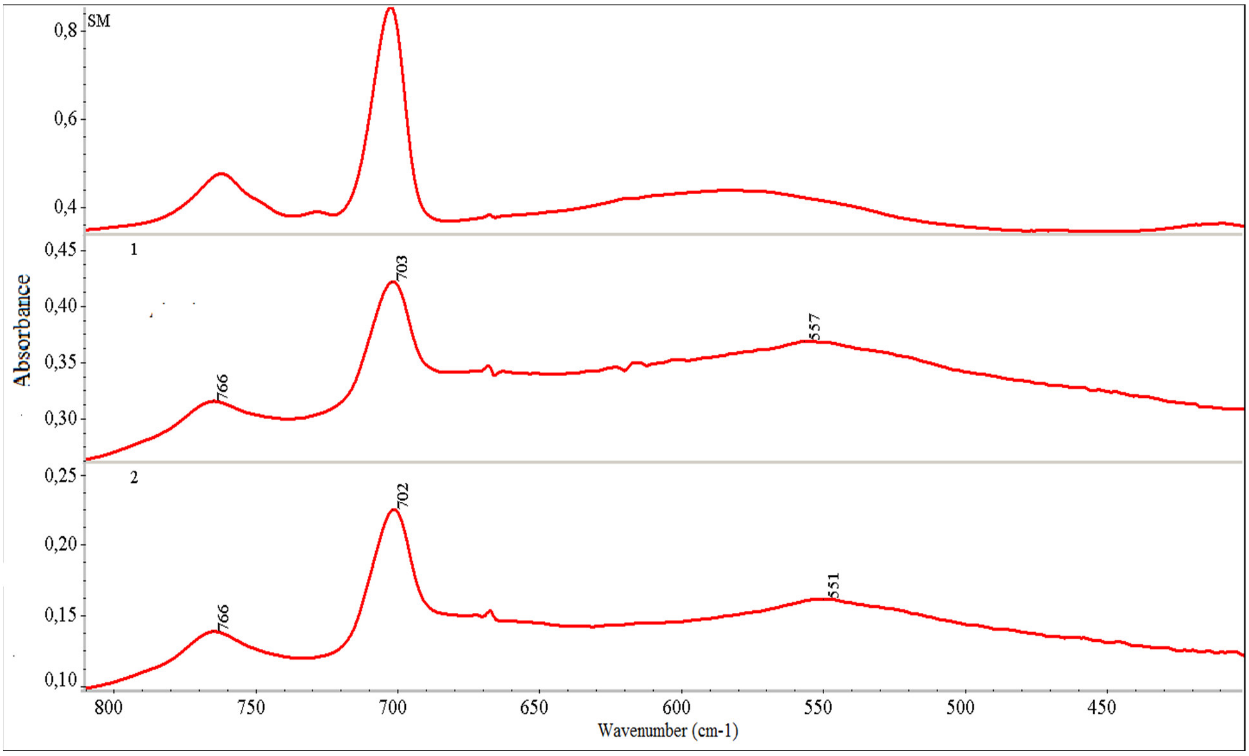

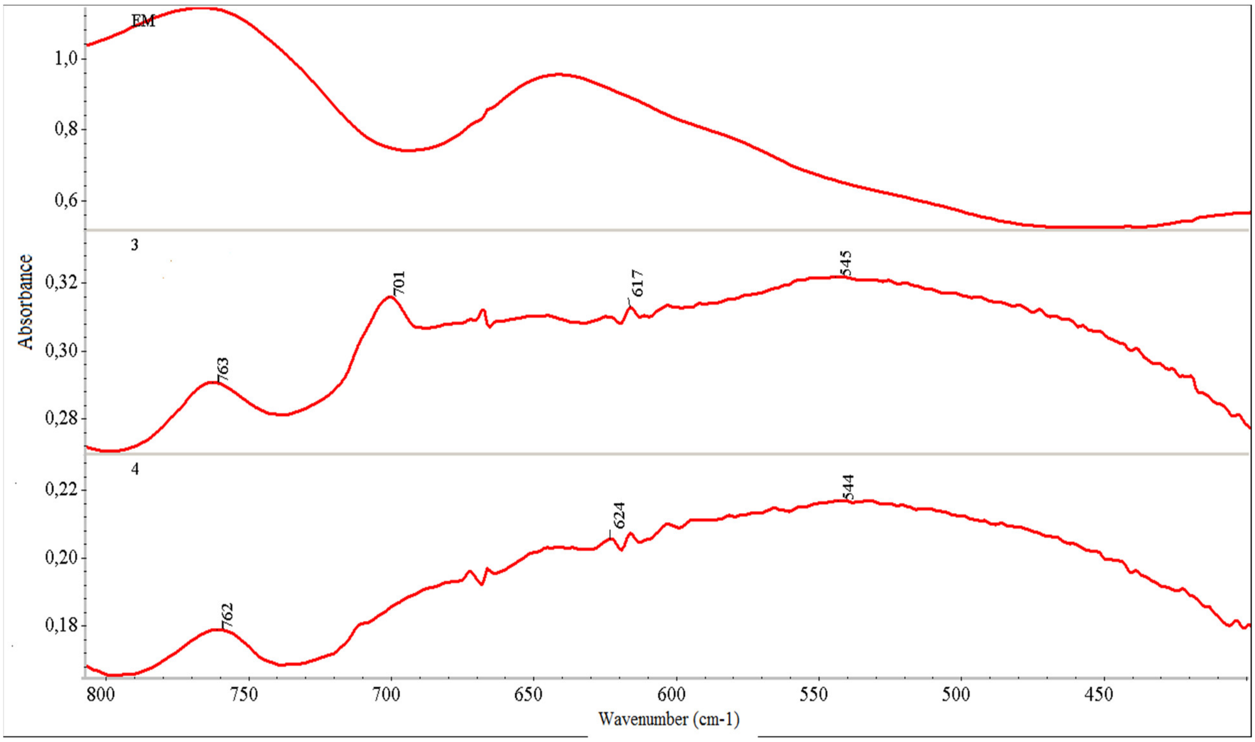

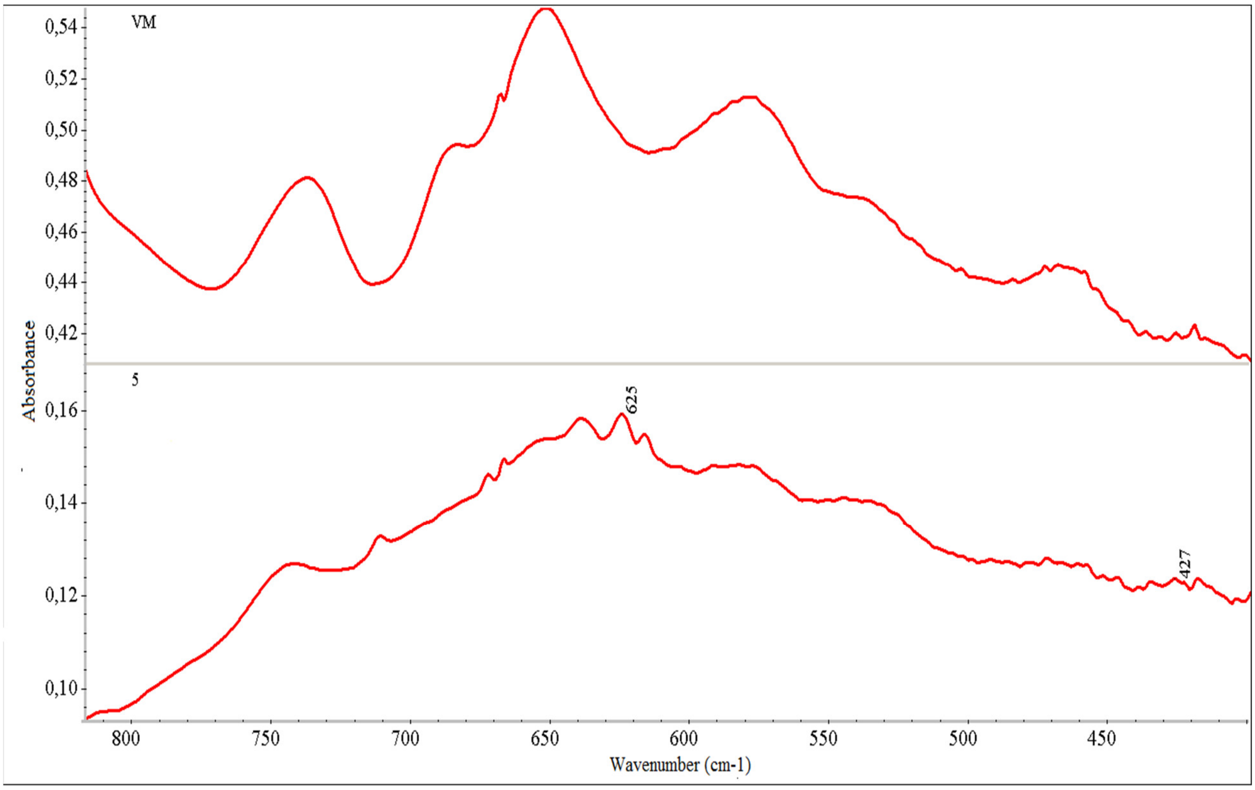

Figure S5. FTIR spectra of samples: SM/Au^0/ZnO (1), SM/Ag^0/ZnO (2), EM/Au^0/ZnO (3), EM/Ag^0/ZnO (4) and VM/Au^0/ZnO (5); SM, EM and VM (initial copolymers).

The survey XPS spectra of heterodimers samples in the form of sodium salts are displayed in Figures S 6-9. The quantification data derived from the survey and high-resolution spectra are presented in Tables S1.

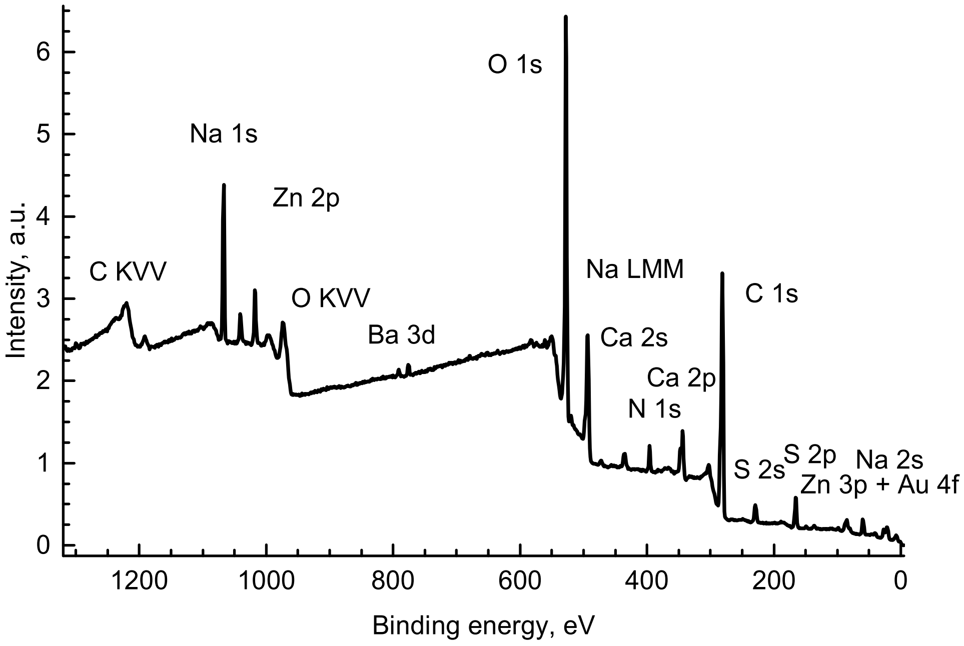

Figure S6. The survey spectrum of the VM/Au^0/ZnO sample.

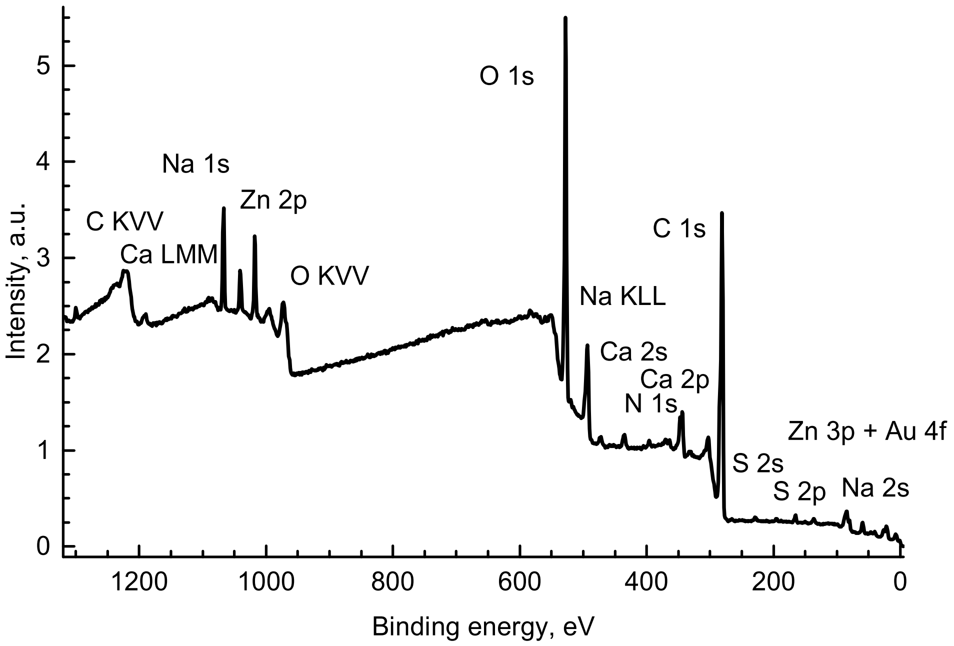

Figure S7. The survey spectrum of the EM/Au^0/ZnO sample.

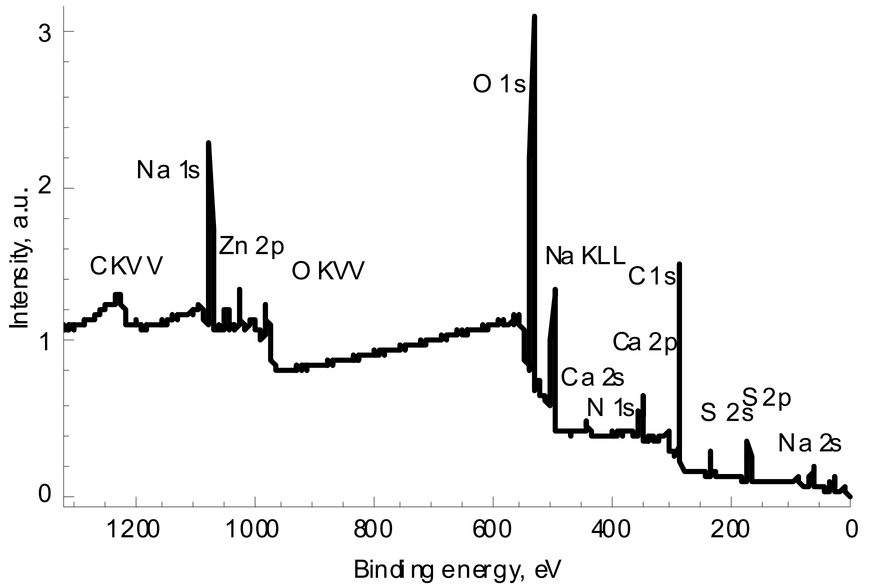

Figure S8. The survey spectrum of the SM/Au^0/ZnO sample.

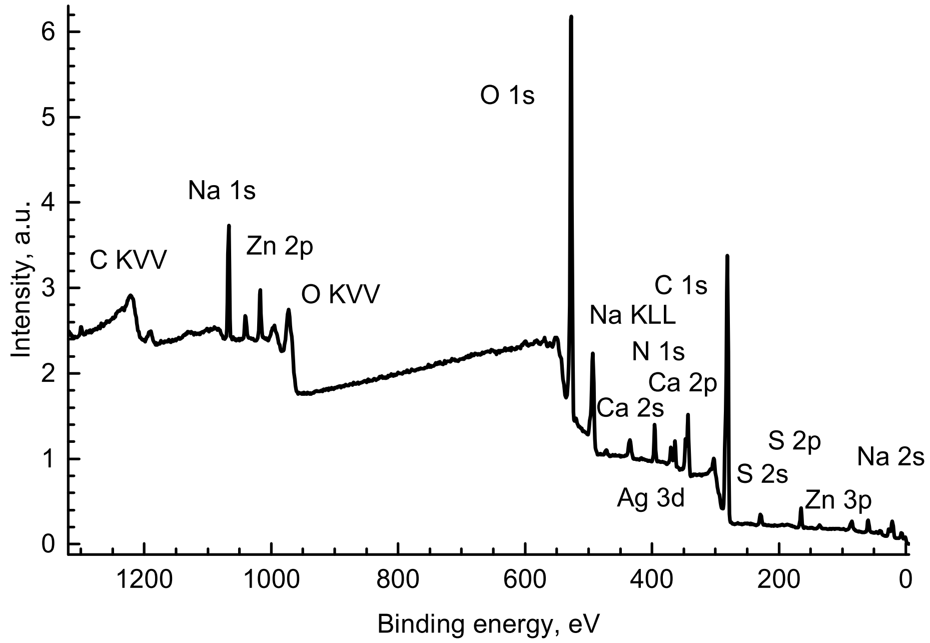

Figure S9. The survey spectrum of the VM/Ag^0/ZnO sample.

Table S1. XPS quantification data (at. %) determined from the high-resolution spectra.

Sample	Zn	Au	Ag	O	C	N
VM/Au^0/ZnO	0.9	0.04		30.6	65.6	2.9
EM/Au^0/ZnO	1.1	0.09		27.5	71.3	
SM/Au^0/ZnO	0.7	0.09		36.4	62.8	
VM/Ag^0/ZnO	0.7		0.4	29.9	65.2	3.8

Figure S10 displays the C 1s spectra of the samples fitted with several Gaussian profiles.

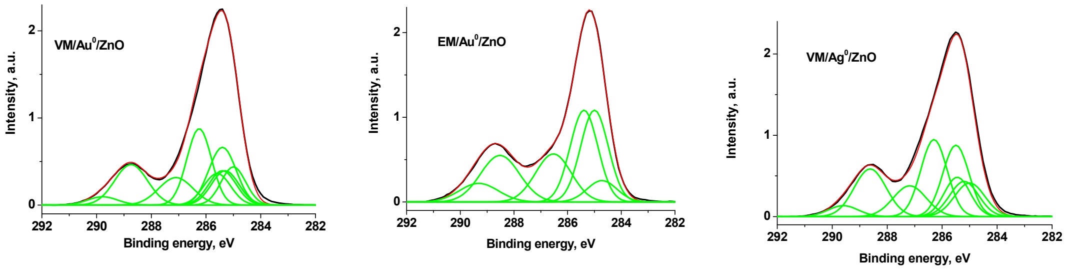

Figure S10. The C 1s high-resolution spectra of the VM/Au^0/ZnO, EM/Au^0/ZnO and VM/Ag^0/ZnO samples.

The fitting is based on the reference chemical shifts [3] taking into account the chemical structures for the samples. Table 3 shows their assignments and characteristics. It should be noted that there are four different C-C/C-H groups: the first is related to low molar mass adventitious carbon (LM, E_b = 284.7 eV), the second is in main aliphatic line non-bonded to C(O)O group (E_b = 285.0 eV), the third is in poly(N-vinylpyrrolidone) ring (E_b = 285.3 eV) and the fourth is related to adventitious carbon which is not in good electric contact with the sample.

In the spectrum of the sample EM/Au^0/ZnO, along with a peak with a binding energy of 284.7 eV, attributed to adventitious carbon, there is also a peak at 286.5 eV, related to C-OH and C-O-C groups. It was previously shown that transition elements supported noble metals are the most effective catalysts in hydrogenation of succinic acid, and when heated without hydrogen in water containing system, butanediol was formed [4].

The N 1s spectra of the VM/Au^0/ZnO and VM/Ag^0/ZnO samples are similar (Figure S 11).

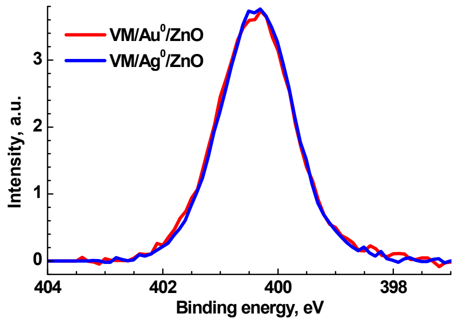

Figure S11. The N 1s high-resolution spectra of the VM/Au^0/ZnO and VM/Ag^0/ZnO samples.

The binding energy of the N 1s peaks (400.4 eV) is 0.5 eV less that of PVNP [3]. This is the reason why the binding energies of the C 1s peaks differ from those of PVNP ring [3].

S12. Calculated data

The standard electrode half - reaction potential of $2H_2O = O_2 + 4H^+ + 4e$ is 1.23 V. Since the synthesis of nanoparticles was carried out at pH = 12 in a boiling aqueous solution, the temperature is about 373K. Consequently, the electrode potential under these conditions can be calculated by equation (1) and is + 0.34 V.

$$\varepsilon_{O_2,\ H^+/H_2O} = \varepsilon^0_{O_2,\ H^+/H_2O} - 0.074pH = +0.34\ \text{B} \quad (1)$$

For the half-reaction $Au(OH)_3 + 3H^+ + 3e = Au^0 + 3H_2O$, the standard electrode potential is +1.45 V, and the electrode potential in the conditions of nanoparticle synthesis can be calculated by equation (2) and is + 0.56 V.

$$\varepsilon_{Au(OH)_3,H^+/Au} = \varepsilon^0_{Au(OH)_3,H^+/Au} - 0.074pH = +0.56\ \text{B}\ (2)$$

Therefore, there are thermodynamic prerequisites for spontaneous oxidation of water under the action of gold (III) hydroxide with the formation of gold nanoparticles.

As to silver, hydroxide and then silver oxide are formed as intermediates under silver nitrate hydrolysis. For the reaction $Ag_2O + 2e + 2H^+ = 2Ag^0 + H_2O$, the standard electrode potential is +1.173 V. Under the conditions of nanoparticle synthesis, the electrode potential calculated by equation (3) is +0.285V.

$$\varepsilon_{Ag_2O,H^+/Ag} = \varepsilon_{Ag_2O,H^+/Ag} - 0.074pH = +0.285\ \text{B} \quad (3)$$

Thus, spontaneous oxidation of water under the action of silver (I) oxide does not occur under the conditions of nanoparticle synthesis. At the same time, the influence of dispersion was not taken into account when conducting thermodynamic calculations. The standard Gibbs energy of Ag_2O formation is - 11.3 kJ/mol. At the same time, the Gibbs energy gain for spherical Ag_2O nanoparticles caused by the curvature of the surface can be calculated using the Kelvin equation (4):

$$\Delta G_d = \frac{4\sigma V_m}{d}\ (4)$$

where: ΔG_d– Gibbs energy gain due to dispersion; σ – surface tension; V_m – molar volume; d – diameter of nanoparticles.

Since for the vast majority of crystals, the surface tension is of the order of 1 J/m^2, and the molar volume of Ag_2O is 3.25×10^{-5} м^3/моль, then with a dimeter of nanoparticles of 10 nm, the value of ΔG_d is + 13 kJ/mol. Consequently, the Gibbs energy of Ag_2O nanoparticles with a diameter of 10 nm, calculated by equation (5), is +1.7 kJ/mol and will increase with decreasing nanoparticle diameter.

$$\Delta G = \Delta G^0 + \Delta G_d\ (5)$$

where: ΔG^0 is the standard Gibbs energy of formation, ΔG is the Gibbs energy taking into account dispersion.

Thus, there are thermodynamic prerequisites for the spontaneous decomposition of Ag_2O nanoparticles with a diameter of about several nanometers and the formation of silver nanoparticles already at a temperature of 298 K. The latter assumption is confirmed by the data given in [5]. As the temperature increases, the tendency to Ag_2O decomposition will increase.

References

1. Megarajan, S.; Kamlekar, R. K.; Kumar, P. S.; Anbazhagan, V. Rapid and selective colorimetric sensing of Au^{3+} ions based on galvanic displacement of silver nanoparticles. *New J. Chem*., **2019**, 43. DOI:10.1039/C9NJ04289J.

Samoilova, N.A.; Krayukhina, M.A. Evaluation of the sorption capacity of a maleic acid copolymer towards gold (III) ions and production of catalytically active gold nanoparticles on its base. *INEOS OPEN*, **2021**, 4, 149–153. DOI: 10.32931/io2119a

2. Said, A.; Sajti, L.; Giorgio, S.; Marine W. Synthesis of nanohybrid materials by femtosecond laser ablation in liquid medium. *J. Physics: Conf. Series,* **2007**, 59, 259–265. doi:10.1088/1742-6596/59/1/055.

3. Beamson, G.; Briggs, D. High Resolution XPS of Organic Polymers: The Scienta ESCA300 Database. John Wiley & Sons; **1992**.

4. Baidya, P. K.; Sarkar, U.; Villa, R.; Sadhukhan, S. Liquid-phase hydrogenation of bio-refined succinic acid to 1,4-butanediol using bimetallic catalysts. *BMC Chem. Eng*. **2019**, 1, 1-12, https://doi.org/10.1186/s42480-019-0010-z.

5. Gallardo, O. A. D.; Moiraghi, R.; Macchione, M. A.; Godoy, J. A.; Pérez, M. A.; Coronado, E. A.; Macagno, V. A. Silver oxide particles/silver nanoparticles interconversion: susceptibility of forward/backward reactions to the chemical environment at room temperature. *RSC Advances*, **2012**, 2, 2923. doi:10.1039/c2ra01044e.
